# The role of AMPK in macrophage metabolism, function and polarisation

**DOI:** 10.1186/s12967-023-04772-6

**Published:** 2023-12-08

**Authors:** Yinxing Cui, Junhua Chen, Zhao Zhang, Houyin Shi, Weichao Sun, Qian Yi

**Affiliations:** 1 Department of Physiology, School of Basic Medical Science, Southwest Medical University, Luzhou, 646000 China; 2 Department of General Surgery, Dongguan Huangjiang Hospital, Dongguan, 523061 Guangdong China; 3 Department of Orthopedics, The Affiliated Traditional Chinese Medicine Hospital of Southwest Medical University, Luzhou, 646000 China; 4 Department of Bone Joint and Bone Oncology, Shenzhen Second People’s Hospital, Shenzhen, 518035 Guangdong China; 5 The Central Laboratory, Shenzhen Second People’s Hospital, Shenzhen, 518035 Guangdong China

**Keywords:** AMPK, Macrophage, Metabolism, Polarisation, Macrophage-related diseases

## Abstract

AMP-activated protein kinase (AMPK) is a ubiquitous sensor of energy and nutritional status in eukaryotic cells. It plays a key role in regulating cellular energy homeostasis and multiple aspects of cell metabolism. During macrophage polarisation, AMPK not only guides the metabolic programming of macrophages, but also counter-regulates the inflammatory function of macrophages and promotes their polarisation toward the anti-inflammatory phenotype. AMPK is located at the intersection of macrophage metabolism and inflammation. The metabolic characteristics of macrophages are closely related to immune-related diseases, infectious diseases, cancer progression and immunotherapy. This review discusses the structure of AMPK and its role in the metabolism, function and polarisation of macrophages. In addition, it summarises the important role of the AMPK pathway and AMPK activators in the development of macrophage-related diseases.

## Introduction

Metabolism provides cells with the energy required to perform their life-sustaining functions [1]. Cells perceive their environment and reprogramme their metabolism accordingly. Changes in energy metabolism and nutrient levels, as well as stimulation of cytokine receptors, may disrupt the balance of various metabolic pathways. AMP-activated protein kinase (AMPK) is a ubiquitous sensor of energy and nutritional status in eukaryotic cells. It plays a key role in regulating cellular energy homeostasis by promoting catabolism and inhibiting anabolism. In addition, it can monitor changes in the AMP:ATP or ADP:ATP ratio. As the AMP:ATP or ADP:ATP ratio increases when energy is insufficient, AMPK restores energy balance by promoting ATP production through catabolic pathways and inhibiting ATP consumption through synthetic metabolic pathways, thereby regulating metabolic stress [2–6]. Owing to its role in regulating metabolism, AMPK has been considered a promising pharmacological target for treating diseases such as diabetes, obesity, cardiometabolic diseases and cancer [7].

Enzymes that regulate or affect cell metabolism play an important role in controlling the development, survival and function of immune cells [8]. In particular, AMPK can adjust multiple metabolic pathways while maintaining energy homeostasis in T cells, macrophages and dendritic cells. It is expressed in immune cells and serves as an important regulator of inflammatory responses. Inflammatory responses are generated through the coordinated activity of numerous cells and soluble mediators. Macrophages play a key role in inflammatory responses, and their metabolic characteristics are closely related to immune-related diseases, infectious diseases, cancer progression and immunotherapy [9, 10]. AMPK is located at the intersection of macrophage metabolism and inflammation. It regulates the polarisation of macrophages toward the anti-inflammatory M2 phenotype through signal transduction [11]. Here, we review the recent research progress on the role of AMPK in macrophage metabolism, function and maintenance.

## Heterotic structure of AMPK

AMPK is a conserved serine–threonine protein kinase complex that belongs to the Ca^2+^/calmodulin-dependent protein kinase (CAMK) family. Mammalian AMPK was first identified in 1973; however, the name AMPK was finally adopted for its allosteric modulator AMP in 1988 [12, 13]. AMPK is a heterotrimeric complex comprising a catalytic subunit, AMPKα, and two regulatory subunits, namely, AMPKβ and AMPKγ [7, 14–17]. In humans, each AMPK complex is composed of one α-subunit, one β-subunit and one γ-subunit. The different subunits of AMPK are encoded by different genes. α1 and α2 subunits are encoded by protein kinase AMP-activated catalytic subunit alpha 1 (PRKAA1) and PRKAA2, respectively. β1 and β2 subunits are encoded by protein kinase AMP-activated non-catalytic subunit beta 1 (PRKAB1) and PRKAB2, respectively. The subunits γ1, γ2 and γ3 subunits are encoded by protein kinase AMP-activated non-catalytic subunit gamma 1 (PRKAG1), PRKAG2 and PRKAG3, respectively. All possible combinations of these subunits generate 12 different AMPK complexes. Although AMPK heterotrimers are commonly expressed, studies have shown significant differences in expression patterns between species and different cell types. For example, AMPKα1β2γ1 is mainly expressed in human liver cells, while AMPKα2β1γ1 is mainly expressed in rodent liver cells [18]. Similarly, subtype γ2 is highly expressed in the human heart, while subtype γ1 appears to be the main culprit in rodent hearts γ Subunits [19]. In addition, certain cell types only contain a subset of these combinations, indicating that certain complexes have specific effects. For example, in muscles, only the complexes α1β2γ1, α2β2γ and α2β2γ3 were activated by Thr172 phosphorylation, even though the complexes were not as abundant as those containing γ1 [20]. AMPKα contains kinase domains and key residues that are phosphorylated by upstream kinases, including Thr174 in the α1 subunit and Thr172 in the α2 subunit. AMPKβ contains a carbohydrate-binding module (CBM), also known as glycogen-binding domain (GDB), which enables the interaction of AMPK with glycogen. AMPKγ contains four tandem cystathionine β-synthase (CBS) motifs that allow AMPK to bind to adenine nucleotides to respond to changes in AMP, ADP and ATP levels [2, 4, 20–22].

## Regulation of AMPK activity

Classical AMPK activation is triggered by events that increase the cellular AMP-to-ATP and/or ADP-to-ATP ratio, such as hypoxia, starvation, administration of certain drugs, certain diseases, glucose deprivation or muscle contraction [5, 6, 23]. AMPK can respond to changes in nutrition and environment by integrating nutrition and hormone signals to maintain cellular energy homeostasis [17]. Most commonly, AMPK is activated through three mechanisms, including promoting phosphorylation of Thr172, inhibition of protein phosphatase dephosphorylation of Thr172, and directing allosteric activation [6, 7, 12, 23–26].

AMPKα contains the kinase domain responsible for the response to energy stress. Phosphorylation of Thr172 is the main event required for the complete activation of AMPK, leading to the transition of AMPK from an inactive form to a catalytic active form [15, 22, 27, 28]. Owing to metabolism, energy stress or response to Ca^2+^ signals, AMPK is activated upon phosphorylation of Thr172 induced by upstream kinases, including liver kinase B1 (LKB1), calmodulin-dependent protein kinase kinase-β (CaMKKβ) and TGF beta-activated kinase 1 (TAK1) [4, 6, 22, 29, 30]. When the cellular concentration of AMP or ADP increases, the latter directly interacts with AMPKγ, leading to the conformational activation of the kinase. These conformational changes in turn protect AMPK from dephosphorylation at the Thr172 residue by protein phosphatase 2 A (PP2A) and PP2C [8, 17, 22–24, 27, 31–34]. It is noteworthy that CaMKKβ can phosphorylate the Thr172 residue of AMPK in response to increased levels of cellular Ca^2+^; however, its effects are not related to any changes in the cellular AMP-to-ATP ratio [16, 17, 23].

**Fig. 1:**
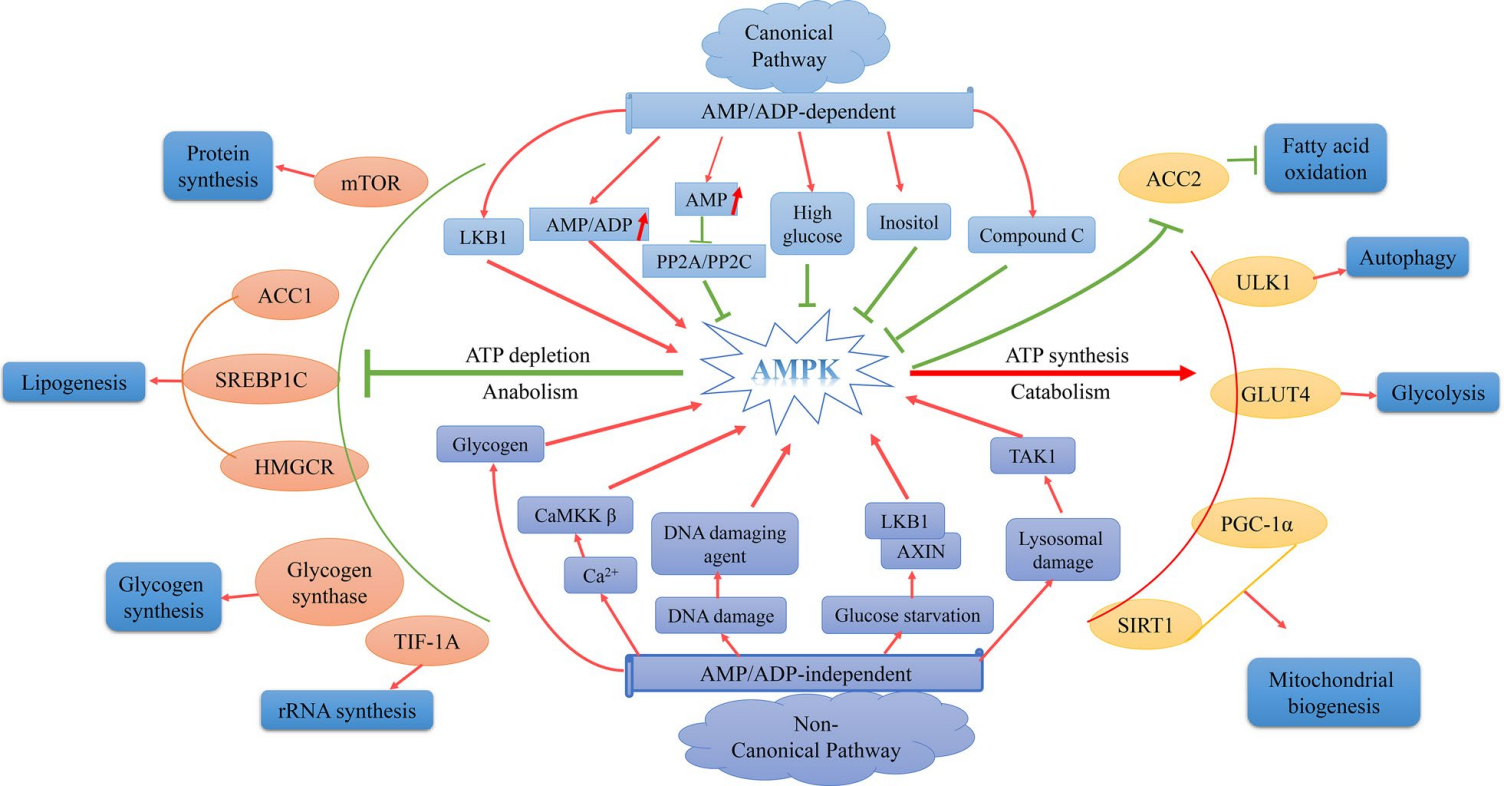
AMPK activation pathways and their regulatory effects on various metabolic processes. AMPK is activated via canonical (AMP/ADP-dependent) and non-canonical (AMP/ADP-independent) pathways. In canonical pathways, AMPK is activated in response to increased levels of AMP or ADP, and the upstream kinase LKB1 phosphorylates AMPK upon activation. AMP/ADP can directly activate AMPK through conformational modification and inhibit PP2A/C-mediated dephosphorylation of AMPK. In non-canonical pathways, AMPK is activated through other mechanisms instead of responding to an increase in AMP/ADP levels. AMPK can respond to an increase in Ca^2+^ levels and is phosphorylated by the upstream kinase CaMKKβ. Under lysosomal damage, the upstream kinase TAK1 activates AMPK. Under glucose starvation, the lysosomal adapter protein AXIN1 or AXIN2 binds to LKB1, leading to the phosphorylation of AMPK. In addition, AMPK can be activated by glycogen, DNA damage agents and AMPK activators. Upon activation, AMPK inhibits ATP-consuming biosynthetic pathways, such as mTOR, ACC1/SREBP1C/HMGCR and TIF-1 A pathways and glycogen synthesis, to inhibit protein synthesis, lipogenesis, glycogen synthesis and rRNA synthesis. In addition, AMPK activates the catabolic pathways that produce ATP, such as ACC2, ULK1, GLUT4 and PGC-1α/SIRT1, to enhance fatty acid oxidation, autophagy, glycolysis and mitochondrial biogenesis. Under normal circumstances, inositol inhibits AMPK activation by binding to AMPK subunits. High glucose and ATP competitive AMPK inhibitor compound C can also inhibit the activity of AMPK. The red arrow is meant to activate. The green arrow is meant to inhibit * LKB1* liver kinase B1, *PP2A/C* protein phosphatase 2 A/C, *CaMKKβ* calmodulin-dependent protein kinase kinase-β, *TAK1* transforming growth factor kinase 1, *AXIN1* axis inhibition protein 1, *SREBP1* sterol regulatory element-binding protein 1, *HMGCR* HMG-CoA reductase, *ACC1* acetyl coenzyme A carboxylase 1, *TIF-1 A* transcriptional intermediary factor 1 A, *mTOR* mammalian target of rapamycin, *PGC-1* peroxisome proliferator-activated receptor-gamma coactivator, *ULK1* UNC-51-like kinase 1, *GLUT4* glucose transporter 4, *SIRT1* sirtuin 1

Given that different AMPK isomers have different locations, downstream targets and upstream kinases, non-standard (i.e. AMP/ADP-independent) activation pathways have attracted substantial interest. These pathways can regulate activity through phosphorylation and conformational changes at the Thr172 residue [6]. For example, certain AMPK activators can directly interact with specific subunits of AMPK, inducing conformational changes and directly leading to AMPK activation, and are not related to changes in cellular ATP, ADP or AMP levels [16, 35–38]. Direct activators can be composed of AMP analogues (competitive activators), simulating the effects of AMP on in vivo AMPK (such as AICAR), or directly interacting with AMPK β Compound with subunit binding (e.g. A769662). Most of these compounds bind together by α and β The specific pockets formed by subunit interactions are called allosteric drug and metabolite (ADaM) sites. The conformational activation of AMPK by binding ligands at the ADaM site greatly stabilizes the kinase domain and prevents dephosphorylation. Indirect activators are composed of compounds that indirectly activate enzymes. They may affect the AMP/ATP ratio, respiratory chain reaction, carbohydrate uptake, or ATP production. These are mainly polyphenols and alkaloids [7, 19, 39]. For example, activation of AMPK by metformin requires the upstream kinase LKB1. AMPK regulates cellular metabolic balance through various direct or indirect activation pathways (Fig. 1). Upon activation, AMPK directly or indirectly regulates the activity of rate-limiting metabolic enzymes, transcription and translation factors and epigenetic regulators through phosphorylation of downstream targets. Subsequently, it readjusts metabolism to promote catabolism or inhibit anabolism, thereby inhibiting cell proliferation and growth (Fig. 1). In particular, AMPK activates catabolic pathways to produce ATP and inactivates ATP-consuming biosynthetic pathways, including glycogen, fatty acid and protein synthesis pathways, which target other signalling pathways, such as sterol regulatory element-binding protein 1/HMG-CoA reductase/acetyl coenzyme A carboxylase 1 (SREBP1/HMGCR/ACC1), transcription initiation factor 1 A (TIF-1 A) and mTORC1 pathways. For example, AMPK enhances glucose/fat uptake, mitochondrial metabolism and cellular autophagy by phosphorylating glucose transporter 4/platelet glycoprotein 4 (GLUT4/CD36), peroxisome proliferator-activated receptor-gamma coactivator lalpha (PGC-1a), sirtuin 1 (SIRT1) and acetyl coenzyme A carboxylase 2 (ACC2), respectively [5, 8, 14, 17, 19, 21, 25, 29, 40–43].

Expressed in macrophages, AMPK acts as an important regulator of inflammatory responses. It can indirectly inhibit the activation of nuclear factor kappa B (NF-kB), a key regulator of innate immunity and inflammation, by modulating the activation of multiple downstream targets including SIRT1, forkhead box protein O (FOXO) and PGC-1α in macrophages [24]. Altogether, AMPK regulates not only cellular metabolism but also cellular functions including autophagy, mitochondrial and lysosomal homeostasis, DNA repair and immunity. In the following sections, we discuss the role of AMPK in the polarisation and metabolic function of macrophages.

## Regulation of macrophage activation

Macrophages are widely distributed innate immune cells. They not only participate in the development and repair of tissues but also prevent pathogenic infection, chronic inflammation, fibrosis and cancer [44]. They are classified as follows: tissue-resident macrophages derived from ancestral cells produced in the yolk sac and macrophages derived from single-core cells from bone marrow-derived haematopoietic stem cells. The role of macrophage is that general tissue macrophages prenatally from yolk are born to subserve homeostatic functions and monocyte-derived cells are involved in response to pathological signals [45]. Macrophages are known by different names in different tissues. For instance, they are called pulmonary macrophages, microglia, osteoclasts, and Kupffer cells in the lungs, nervous system, bone, and liver respectively [46]. The involvement of macrophages in multiple biological processes suggests that they can adequately adapt to their microenvironment and have unusual plasticity. Macrophages can be polarised by microenvironmental stimuli and signals to perform specific functions [44].

Macrophages are characterised by their classic (M1) and alternative (M2) phenotypes. Stimulation with lipopolysaccharides (LPSs) and interferon-gamma (IFN-γ) induces the polarisation of macrophages toward an inflammatory phenotype commonly referred to as the M1 phenotype. Activation of macrophages by interleukin 4 (IL-4) ,IL-10, and IL-13, induces their polarisation toward an anti-inflammatory phenotype commonly referred to as the M2 phenotype [9, 47–51].

**Fig. 2:**
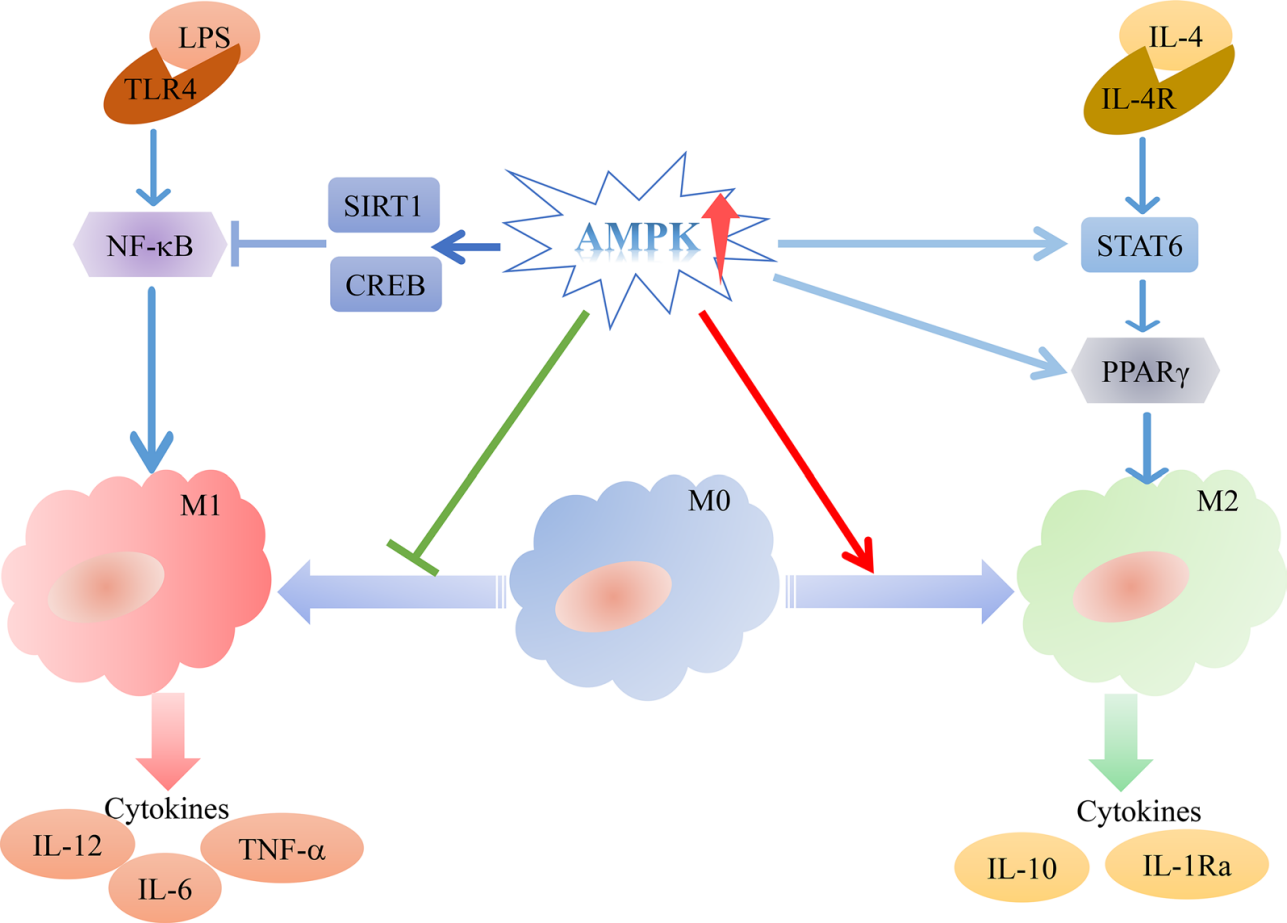
AMPK is the important trigger for macrophage polarisation. On the one hand, LPSs promote M1 polarisation through the NF-κB pathway and the secretion of inflammatory factors such as IL-12, IL-6 and TNF-α. On the other hand, activation of AMPK inhibits the NF-κB pathway by upregulating the expression of SIRT1 and CREB, thereby inhibiting M1 polarisation. AMPKα-knockout macrophages display M1 hyperpolarisation. IL-4 promotes the polarisation of M2 macrophages by activating the downstream STAT6/PPARγ pathway and the secretion of the anti-inflammatory factors IL-10 and IL-1Ra. STAT6 is akey transcription factor involved in IL-4-mediated M2 polarisation. On the other hand, activation of AMPK promotes the upregulation of STAT6 and PPARγ, thereby affecting the polarisation of M2 macrophages. *LPS* lipopolysaccharide, *TLR* toll-like receptor, *SIRT1* sirtuin 1, *CREB* cAMP-response element-binding protein, *NF-κB* nuclear factor κB, *IL* interleukin, *IL-4R* interleukin-4 receptor, *PPAR* peroxisome proliferator-activated receptor, *STAT* signal transducers and activators of transcription, *TNF* tumour necrosis factor

M1 macrophages have high microbicidal activity and enhance the production of ROS and pro-inflammatory cytokines, such as IL-1β and tumour necrosis factor alpha (TNF-α). They can not only promote the clearance of foreign pathogens and tumour cells but also impede wound healing and tissue regeneration by mediating ROS-induced tissue damage [52]. On the contrary, M2 macrophages participate in long-term tissue repair and protect against extracellular parasites [53]. Macrophages can alter their phenotype in response to new environmental stimuli after polarisation. This reversibility of polarisation holds important therapeutic value, especially in diseases in which M1/M2 imbalance plays a pathogenic role [54, 55]. Macrophage polarisation is driven by changes in the tumour microenvironment, nanocarriers, phagocytosis and different disease-related factors [51]. Therefore, elucidating the signalling pathways that regulate macrophage polarisation may facilitate the development of strategies for modifying macrophage behaviour. Macrophage polarisation involves the regulation of multiple signalling pathways and transcriptional networks, including NF-κB, peroxisome proliferator-activated receptor-gamma (PPAR-γ), phosphatidylinositol 3-kinase/protein kinase B (PI3K/AKT), MAPK, Janus kinase/signal transducer and activator of transcription (JAK/STAT), Wnt/β-catenin and AMPK signalling pathways [56]. Given that AMPK is the important trigger for macrophage polarisation, we have discussed the role of only the AMPK signalling pathway in this review [47, 51, 55, 57–63] (Fig. 2).

## Role of AMPK in macrophage polarisation and anti-inflammation

As mentioned above, AMPK acts as an energy receptor that, once activated, inhibits energy-dissipating pathways (e.g., fatty acid synthesis) and affects energy-producing pathways (e.g., glycolysis and glycogenolysis). M1/M2 macrophage activation includes well-coordinated changes in signaling events and post-translational mechanisms, as well as extensive metabolic remodeling. Changes in cell metabolism determine their different physiological behaviors. In terms of glucose metabolism, M1 macrophages have higher glycolytic activity, while M2 macrophages are more dependent on oxidative phosphorylation [64]. AMPK, while AMPK stimulates glycolysis, involving phosphorylation of phosphofructokinase 2 (PFK-2) at Ser466. PFK-2 catalyzes the conversion of fructose-6-phosphate to fructose-1,6-bisphosphate, which is the third stage of glycolysis. In terms of lipid metabolism, the regulation of fatty acid synthesis (FAS) and fatty acid oxidation (FAO) drives macrophage M1/M2 activation, respectively. AMPK blocks cholesterol synthesis and indirectly regulates the activity of FAS [39]. In terms of amino acid metabolism, amino acid metabolism is also an important way to maintain the immune activity of macrophages. Since protein synthesis is a high-energy-demanding process, the energy-sensing AMPK signaling pathway is linked to mTOR and can indirectly affect the amino acid metabolism process of macrophages [65].

Therefore, AMPK plays a crucial role in macrophage metabolism and maintaining immune activity. In addition, activation of AMPK is necessary for the efficient polarisation of M1 macrophages to M2 macrophages. AMPK acts as a direct upstream signalling molecule in the initiation of anti-inflammatory signalling pathways in macrophages [10, 24]. Genetic defects in AMPKβ1 in mouse macrophages have been shown to increase the levels of pro-inflammatory cytokines (such as TNF-α, IL-6, monocyte chemotactic protein-1[MCP1] and IL-1β) and decrease the levels of anti-inflammatory cytokines (such as IL-10) [66]. AMPKα1 is the predominant AMPKα isoform expressed by macrophages [47]. Genetic defects in AMPKα1 in macrophages prevent the acquisition of the M2 phenotype in vitro and in vivo and enhance the LPS-induced proinflammatory function of macrophages. However, the constitutive expression of AMPKα1 results in decreased production of pro-inflammatory cytokines and increased production of IL-10 in macrophages [24, 47, 67, 68]. AMPKα1 plays an essential role in activating IL-10 during PI3K/AKT/mTORC1 and STAT3-mediated macrophage polarisation [58]. Rapid activation of AMPK indicates that it acts as a direct upstream signalling molecule in the initiation of anti-inflammatory signalling pathways [58]. When macrophages are activated by IL-4 and IL-13, phosphorylation of AMPK leads to upregulation of PPARδ and angiotensin converting enzyme (ACE), resulting in the inhibition of M1 macrophages and upregulation of cytokines in M2 macrophages [46]. These findings validate that AMPK is a key immunometabolic regulatory factor in macrophages [69]. Some studies have reported that the anti-inflammatory activity of AMPK in macrophages is associated with decreased IκB degradation, enhanced AKT activity and inactivation of glycogen synthase kinase 3 beta (GSK3-β). Inhibition of GSK3-β enables cAMP response element-binding protein (CREB) to compete for the nuclear coactivator CREB-binding protein (CBP) required for the function of NF-κB, thereby reducing the expression of pro-inflammatory genes [24, 47, 58]. Many direct activators of AMPK have been shown to inhibit the inflammatory function and promote the anti-inflammatory function of macrophages [70]. For example, treatment with the AMPK agonist metformin can reduce the production of pro-inflammatory cytokines in bone marrow-derived macrophages (BMDMs). Therefore, AMPK plays an important role in promoting the anti-inflammatory phenotype of macrophages [66, 68]. In response to infection, toll like receptor 4 (TLR4) on macrophages recognises LPSs to induce the expression of FMS related tyrosine kinase 4 (FLT4). After identification of LPS or bacterial infection, TLR4 initiates the MyD88-dependent pathway for NF-κB activation. TLR4-MyD88-NF-κB signaling increases the expression level of FLT4 and its ligand VEGFC in bacteria-infected macrophages, which in turn forms a feedback loop to inhibit TLR4-induced inflammatory responses in macrophages [71]. In addition, AMPK directly or indirectly regulates various aspects of autophagy mechanisms in response to turnover of old or damaged molecules, supplementation of nutrient storage during starvation, and removal of intracellular pathogens. AMPK is the main activator of autophagy during oxidative stress and energy starvation catabolism [20, 72]. FLT4 activates AMPK to prevent caspase 1-induced pyroptosisand excessive inflammation and enhances phagocytosis and autophagy to clear bacteria. The AMPK agonist 5-Aminoimidazole-4-carboxamide ribonucleotide (AICAR) can salvage glycolytic reprogramming and inflammasome activation in macrophages expressing mutant FLT4 to prevent recurrent infections [73].

AMPK not only directs metabolism in macrophages but also plays a crucial role in determining the inflammatory state of macrophages [68]. As described earlier, AMPK is a potent negative regulator of the inflammatory function of macrophages and promotes the polarisation of macrophages toward an anti-inflammatory phenotype. Some studies have demonstrated that AMPK is actively involved in the regulation of the phagocytic function of macrophages. Treatment of macrophages with AMPK activators results in cytoskeletal rearrangement and morphological changes, including increased cell size, and enhances the phagocytic function of the enlarged macrophages [74]. Macrophages are key cells that regulate the regression of inflammation after tissue injury. Glucocorticoids (GCs) are the most effective anti-inflammatory hormones in clinical use. On the one hand, AMPK is necessary to promote repair of macrophage phenotypes. On the other hand, glucocorticoids activate AMPKα1 in macrophages, thereby inducing the acquisition of a repair phenotype and promoting the resolution of inflammation in vivo during muscle regeneration after injury and acute lung injury [75]. This suggests that AMPK not only promotes M2 polarization, but also plays an important role in injury resolution through metabolic to transcriptional regulation of regenerative inflammation.

## Role of AMPK in macrophage-related diseases

AMPK plays an important role in macrophage-related diseases. Metabolism in macrophages is closely related to immune-related diseases, infectious diseases, cancer progression and immunotherapy [9, 76]. For example, M2-polarised tumour-associated macrophages (TAMs) play an important role in tumour progression and metastasis, demonstrating their potential as an attractive therapeutic target [77]. AMPK activators can alleviate macrophage-related diseases through AMPK-related pathways (Table 1). The following section describes the role of AMPK in macrophage-related conditions and diseases, such as atherosclerosis, insulin resistance and cancer.

### Atherosclerosis

As early as 1973, AMPK activity was found to be associated with HMGCR and ACC, which are key regulators of cholesterol and fatty acid synthesis. It regulates lipid metabolism by directly phosphorylating proteins or regulating gene transcription in specific tissues, such as the liver, fat and muscle, and reduces the accumulation of inflammatory lipid intermediates, thereby delaying the progression of atherosclerosis [22]. Atherosclerosis is a chronic inflammatory disease. Atherosclerotic plaques are characterised by the accumulation of macrophages (called ‘foam cells’) enriched with cholesterol and lipid, indicating that macrophages are involved in the progression of atherosclerosis. Macrophages have high plasticity and can alter their phenotype in response to an atherosclerotic microenvironment. Therefore, targeting macrophages is a potential therapeutic strategy for atherosclerosis [78–81]. Although both M1 and M2 macrophages are involved in human atherosclerosis, M1 macrophages primarily participate in the progression of atherosclerotic plaques [82]. In addition to lipid metabolism disorder, which leads to atherosclerosis, inhibition of autophagy enhances the production of reactive oxygen species and reduces exocytosis. These consequences lead to the expression of genes that promote inflammation in macrophages and the recruitment of circulating monocytes, contributing to the progression of atherosclerotic plaques [83].

In the early stages of atherosclerosis, activation of AMPK restores cholesterol homeostasis in macrophages by inhibiting the formation of foam cells. C-C motif chemokine receptor 2 (CCR2) is a key factor involved in the accumulation of macrophages during the progression of atherosclerosis. It controls the migration of inflammatory Ly6C^hi^ monocytes from the bone marrow (BM) into the circulating blood. Activation of AMPK prevents this migration by inhibiting the expression of CCR2, thereby reducing the number of Ly6C^hi^ monocytes in blood and atherosclerotic plaques and the accumulation of macrophages in the plaques [84]. Metformin can regulate the function of macrophages in atherosclerosis through the AMPK signalling pathway. It suppresses the differentiation of monocytes and the inflammatory function of macrophages, alleviates oxidative stress and inhibits foam cell formation, thereby delaying the progression of atherosclerosis [79]. In ApoE-knockout (ApoE−/−) mice, AMPKα1 activated during monocyte differentiation promotes autophagy-mediated differentiation and survival of monocytes, thereby increasing the number of macrophages. This phenomenon indicates that AMPKα1 promotes the occurrence and progression of atherosclerosis in ApoE−/− mice [85].

Inflammation is one of the main causes of atherosclerosis. Activation of AMPK not only promotes the transformation of macrophages from a pro-inflammatory (M1) phenotype to an anti-inflammatory (M2) phenotype but also enhances the oxidative metabolism of fatty acids, thereby reducing the accumulation of intracellular lipid metabolites that lead to inflammation [86]. Given that AMPK is an effective counter-regulator of the inflammatory function of macrophages and promotes the polarisation of macrophages toward the anti-inflammatory phenotype, it plays a crucial role in the progression of atherosclerosis. Downregulated AMPK contributes to the persistence of atherosclerosis by increasing inflammation, enhancing lipid synthesis and reducing cholesterol efflux in macrophages [83, 87, 88]. Curcumin enhances cholesterol efflux by activating AMPK-SIRT1-LXRα signal transduction in THP-1 macrophage derived foam cells and up regulating the expression of ATP-binding cassette transporter 1 (ABCA1) [89]. Balasubramide derivative 3 C inhibits JAK2-STAT1 signal transduction and downstream STING-IRF3 activation in macrophages in an AMPK dependent manner, and effectively reduces the atherosclerotic plaque load of HFD fed ApoE mice [90]. Therefore, AMPK can also inhibit the formation of atherosclerosis by reprogramming the lipid metabolism of macrophages.

The LKB1/AMPK/mTOR pathway can enhance autophagy, inhibit lipid synthesis and promote fatty acid oxidation [91]. AMPK induces autophagy directly by activating UNC-51-like autophagy-activating kinase 1, Beclin-1 and VPS34 phosphorylation, and indirectly by targeting the AMPK/mTOR/uncoordinated 51-like kinase 1 (ULK1) pathway [20]. This pathway inhibits the phosphorylation of mammalian target of rapamycin (mTOR)and ULK1 through activation of AMPK and enhances LC-3II accumulation and P62 degradation. AMPK directly activates ULK1 and suppresses the inhibitory effects of mTORC1 on ULK1, thereby enhancing macrophage autophagy [72, 92]. This function of AMPK is critical for handling metabolic substrates and removing damaged or senescent organelles. In addition, it facilitates the flow of free cholesterol out of foam cells and reverses cholesterol transport in atherosclerosis [93]. AMPK not only affects cholesterol efflux through phosphorylation and activation of activating transcription factor 1 (ATF1) but also induces the LXRβ-LXRα regulatory cascade that eventually activates ABCA1 expression and increases cholesterol efflux to inhibit the formation of foam cells. Altogether, AMPK acts as an important regulator of cholesterol homeostasis in human macrophages [78, 86, 94–96].

### Insulin resistance

Insulin resistance, a major complication of diabetes, is a condition in which insulin-induced glucose uptake is impaired in insulin-sensitive tissues. It is considered a reduced response of peripheral tissues to insulin [97]. Insulin resistance in obesity is mainly caused by continuous caloric absorption exceeding caloric expenditure, and Obesity-induced chronic inflammation is an important pathogenic mechanism underlying insulin resistance. Therefore, recruitment of adipose tissue macrophages (ATMs) and activation of pro-inflammatory factors are important for the development of insulin resistance. Compared with lean individuals, obese individuals have a high proportion of total macrophages, with a low proportion of M2 macrophages, in adipose tissues, which aggravates local and systemic insulin resistance [98]. Macrophages are important participants in the progress of diabetes, and promote inflammation by releasing proinflammatory cytokines and proteases [99]. In obesity, the increased levels of proinflammatory cytokines, such as IL-1β, IL-6, TNF-α and MCP1, in adipose tissues area direct cause of insulin resistance. Inflammatory cytokines impair insulin signalling and trigger insulin resistance [100]. LPSs can induce inflammation in fat cells by increasing TNF-α and MCP-1 levels, thereby inducing insulin resistance. Activation of AMPK prevents the LPS-induced M1 polarisation of macrophages, thereby alleviating inflammation and subsequently correcting insulin resistance [101]. These findings indicate that the anti-inflammatory effects of AMPK on macrophages protect the biological characteristics of adipose tissue [102]. Therefore, inhibiting chronic inflammation is a promising therapeutic strategy for improving insulin resistance. In the context of energy homeostasis, insulin resistance is the result of excess energy in cells. AMPK regulates energy balance and is a major sensor of glucose uptake, fatty acid oxidation and mitochondrial biogenesis. In the case of energy surplus, a decrease in AMPK activity leads to the downregulation of GLUT4 activity, which inhibits glucose uptake in insulin resistance and eventually leads to elevated blood sugar levels in patients with obesity and type 2 diabetes mellitus [103]. It is noteworthy that metformin, the most important AMPK activator, is the most common drug used for the treatment of type 2 diabetes mellitus [104]. On the one hand, metformin activates AMPK, thereby inhibiting liver gluconeogenesis and increasing glucose utilization in peripheral tissues [105]. On the other hand, activation of AMPK by metformin can inhibit abnormal extracellular matrix remodeling in adipose tissue and improve insulin resistance in obesity [106], and can exert anti-inflammatory effects by inhibiting intracellular fatty acid synthesis in macrophages [107–110]. In addition, AMPK inhibits inflammatory signalling in macrophages and drives the transformation of proinflammatory M1 macrophages to anti-inflammatory M2 macrophages, which are crucial for chronic inflammation-induced insulin resistance [111]. In macrophage-adipocyte co-culture systems, inactivation of AMPKα1 has been shown to inhibit insulin signalling and glucose uptake and upregulate JNK phosphorylation in adipocytes, indicating the activation of the JNK pathway, which is a key inflammatory pathway leading to insulin resistance [57, 70]. Consistently, in vivo studies have demonstrated that mice lacking AMPKβ1 in haematopoietic cells exhibit the loss of AMPK activity in macrophages, accompanied by the increased accumulation of M1 macrophages in the liver and accelerated development of insulin resistance in response to diets that promote obesity [70, 112]. Furthermore, dihydromyricetin can alleviate inflammation-induced insulin resistance through the phospholipase C (PLC)–CaMKK–AMPK signalling pathway [100], and vaccarin can alleviate insulin resistance and steatosis by activating the AMPK signalling pathway [113]. In mice fed a high-fat diet (HFD), administration of l-lactic acid at moderate doses inhibits the M1 polarisation of ATMs by activating the GPR132–PKA–AMPKα1 signalling pathway, thereby alleviating insulin resistance [111]. Altogether, AMPK-mediated inflammation in macrophages plays an important role in promoting insulin resistance in host tissues.

### Cancer

Dysregulation of cellular energy is a key hallmark of cancer. AMPK activation regulates energy levels and influences cell growth by regulating the targets of the mTOR pathway. Therefore, metabolism-related factors, such as LKB1, which is the major upstream kinase of AMPK, have been identified as tumour suppressors in various malignant tumours [114, 115]. One of the characteristics of cancer cells is aerobic glycolysis, also known as the “Warburg effect”. It is an important component of cancer metabolism reprogramming and a core factor in cancer progression [116]. As a metabolic sensor responsive to low nutrient utilization, AMPK plays a very important role in tumor aerobic glucose metabolism. On the one hand, AMPK plays an important role in promoting cancer cell glycolysis by regulating enzymes such as phosphofructose kinase (PFK). On the other hand, AMPK also enhances glucose uptake in cancer cells by increasing the expression of glucose transporters (GLUT) [117]. According to reports, the Warburg effect is negatively regulated by AMPK. For example, gene ablation of AMPK can lead to normal stability of oxygen content in HIF1, thereby altering the metabolic pathway of aerobic glycolysis and accelerating tumor growth [118]. In addition, inflammation has been recently identified as a marker and transmitter of tumours [119]. Owing to its role in energy regulation and inflammation, AMPK has emerged as an important therapeutic target for cancer [120–125]. Notably, AMPK acts as a tumour suppressor before cancer has developed. However, it inhibits or promotes cancer after its onset based on the cell type or state [102, 126, 127]. Under nutrient deficient conditions, the activation of AMPK makes tumor cells more resistant to metabolic stress due to stress caused by glucose consumption or hypoxia, thereby exhibiting a tumor promoting effect. In the case of sufficient nutrition, AMPK exhibits tumor inhibitory effects [128]. AMPK mediated changes in cancer cell metabolism or tumor microenvironment components to inhibit cancer progression will provide significant therapeutic benefits [129].

Macrophages, one of the most abundant innate immune cells in the tumour microenvironment, are responsible for driving antitumour immunity through phagocytosis and antigen presentation [128]. Macrophage polarisation is one of the prominent events following leukocyte infiltration into the tumour microenvironment. M1 macrophages have proinflammatory and toxic effects on tumour cells, whereas M2 macrophages facilitate tumour progression by inhibiting inflammation and promoting angiogenesis [130]. TAMs, particularly M2-like TAMs, have been associated with a poor prognosis in various types of cancers in clinical and animal studies [82, 131, 132]. Therefore, interfering with the function of M2 TAMs represents a promising strategy for developing novel immunotherapies. Multiple studies have focused on developing strategies aimed at influencing tumour growth and metastasis by driving the transformation of pro-tumoral M2 TAMs to the anti-tumoral M1 phenotype without consuming the entire TAM population [82]. As mentioned above, M1 macrophages have higher glycolytic activity, while M2 macrophages are more dependent on oxidative phosphorylation. So in the aerobic glycolysis of tumor cells, the glucose metabolism and polarization of macrophages contribute significantly to the tumor microenvironment [133]. The subtypes of AMPK are expressed differently in various cancers. These subtypes can manifest as tumor suppressors or promoters, depending on the environment [117]. The two subtypes of AMPKα, AMPKα1 and AMPKα2, play different roles in regulating tumour development. In a study, AMPKα2 knockout was found to enhance tumour growth and liver injury in a mouse model of liver metastasis of colon cancer. AMPKα2 deficiency intensifies inflammatory cell infiltration in tumour tissue and enhance the recruitment and differentiation of M2 macrophages. In addition, it exacerbates tumour development by affecting the inflammatory microenvironment of tumours in liver tissues. However, AMPKα1 is highly expressed in macrophages, and enhanced activation of AMPKα1 in the tumour microenvironment increases M2 polarisation, which promotes tumour progression [119]. Metformin promotes the expression of M1-related cytokines and decreases the expression of M2-related cytokines in cancer cells through AMPK–NF-κB signalling. This phenomenon suggests that metformin attenuates the polarisation of cancer cells toward the M2 phenotype by inhibiting the expression of M2-induced cytokines [134]. Studies have demonstrated that metformin-induced activation of AMPK and subsequent inhibition of mTOR decrease the proportion of myeloid-derived suppressor cells (MDSCs) and M2 macrophages by downregulating the mevalonate pathway [135]. It is noteworthy that metformin induces the formation of the M2 phenotype in monocultured macrophages but attenuates the M2 phenotype and inhibits the expression of M2-related cytokines when macrophages are co-cultured with tumour cells. Therefore, the regulation of macrophage polarisation by metformin depends on the microenvironment, suggesting that the reversibility of macrophage polarisation and their ability to change continuously in response to new environmental stimuli have important therapeutic value in tumours. Astragalus IV (AS-IV) can inhibit the progression and metastasis of lung cancer by blocking M2 polarisation through the AMPK signalling pathway. The percentage of M2 macrophages in tumor tissue treated with AS-IV decreased, and AS-IV inhibited AMPKα activation in M2 macrophages. When knocking down AMPKα in macrophages, the inhibitory effect of AS-IV is partially eliminated, indicating that it inhibits the AMPK signaling pathway in macrophages [77]. Under hypoxic conditions, tumour-derived exosomes induce the polarisation of M2 macrophages through PKM2/AMPK to promote progression of lung cancer [130]. Altogether, the regulation of the AMPK signalling pathway may interfere with tumour progression by altering the phenotype of macrophages and influencing the inflammatory microenvironment of the tumour.

**Table 1:** AMPK activators affect macrophage-related diseases through the AMPK pathway

AMPK enhancers	Experimental design	Applications/outcomes of the study	References
*Astragalus membranaceus*	Model: murine ANA-1 macrophages	Injection of *Astragalus membranaceus* extracts suppressed the production of interleukin-6 by activating autophagy through the AMPK–mTOR pathway in lipopolysaccharide-stimulated macrophages	[136]
Calcium-binding protein 39	Model: murine primary chondrocyte cell line (ATDC5) and macrophage cell line (RAW264.7)	Overexpression of calcium-binding protein 39 promoted macrophage polarisation from the ‘M1’ to the ‘M2’ phenotype and alleviated chondrocyte damage in osteoarthritis by activating the AMP-activated protein kinase/sirtuin-1 axis	[137]
Moderate l-lactate	Model: male C57BL/6 mice fed a high-fat diet	Moderate administration of l-lactic acid inhibited the M1 polarisation of adipose tissue macrophages by activating the GPR132–PKA–AMPKα1 signalling pathway to alleviate insulin resistance	[111]
2-Deoxy-d-glucose (2-DG)	Model: bone marrow-derived macrophages (BMDMs) from C57BL/6 (B6) mice	2-DG treatment decreased M2 polarisation in mice with tumours and allergic airway inflammation via the AMPK–Hif-1α pathway	[138]
Non-lethal sonodynamic therapy (NL-SDT)	Model: Mouse model of AS and bone marrow transplantation (BMT)	Non-lethal sonodynamic therapy facilitated the M1-to-M2 transition in advanced atherosclerotic plaques by activating the ROS–AMPK–mTORC1–autophagy pathway	[59]
β-Hydroxyisovalerylshikonin (β-HIVS)	Model: murine macrophage cell line (RAW264.7) and BMDMs	β-HIVS inhibited M1 polarisation and promoted M2 polarisation through the AMPK/Nrf2 pathway, alleviating sepsis in mice	[139]
Human recombinant annexin A1 (hrANXA1)	Model: male C57BL/6 mice, alx/fpr2/3GFP/GFP mice, AnxA1−/− mice and BMDMs	AnxA1 promoted macrophage skewing to accelerate muscle regeneration through the AANXA1/FPR2/AMPK axis	[10]
Vitamin B6 (VitB6)	Model: wild-type (WT) B129 mice and DOK3-knockout (DOK3−/−) mice	VitB6 inhibited macrophage activation through the AMPK–DOK3 pathway to prevent LPS-induced acute pneumonia in mice	[140]
Metformin	Model: adult male ICR mice, SRA1-KO mice and BMDMs	Metformin decreased plasma HMGB1 accumulation and attenuated CIPN via the AMPK/p38/SR-A1 signalling pathway	[141]
Astragaloside IV	Model: the human lung cancer cell lines A549 and H1299, the human monocyte cell line THP-1 and Lewis lung cancer (LLC) cells	AS-IV inhibited lung cancer progression and metastasis by blocking M2 polarisation partially through the AMPK signalling pathway	[77]
Ac2-26, a pharmacophore N-terminal peptide of ANXA1	Model: male C57BL/6J mice with tMCAO/R and murine BV2 microglial cells	Annexin A1 protected against cerebral ischaemia–reperfusion injury by modulating microglia/macrophage polarisation via the FPR2/ALX-dependent AMPK–mTOR pathway	[142]
Alginate oligosaccharides (AOSs)	Model: murine RAW264.7 cells and BMDMs	AOSs inhibited LPS-mediated inflammatory responses and attenuated dextran sodium sulphate (DSS)-induced colitis by activating AMPK signalling and suppressing NF-κB activation	[143]
Apoptotic extracellular vesicles (ApoEVs)	Model: mouse bone marrow MSCs and BMDMs	ApoEVs inhibited the polarisation of macrophages to the proinflammatory phenotype via the AMPK/SIRT1/NF-κB pathway and suppressed the formation of adjacent osteoclasts by reducing the secretion of TNF-α	[144]
Metformin	Model: 10-week-old male C57BL/6J mice and bone marrow-derived macrophages (BMMs)	Metformin attenuated osteoclast-mediated abnormal subchondral bone remodelling and alleviated osteoarthritis via the AMPK/NF-κB/ERK signalling pathway	[145]
2-DG	Model: male Sprague-Dawley (SD) rats and RAW264.7 macrophages	2-DG promoted macrophage polarisation from the M1 to the M2 phenotype via the AMPK/NF-κB pathway to ameliorate adjuvant-induced arthritis	[146]
Baicalein	Model: HUVECs (human), RAW264.7 macrophages (murine) and MOVAS (murine)	Baicalein targeted the inflammation-associated AMPK/Mfn-2/MAPK signalling pathway to exert anti-atherosclerotic effects	[147]
Mogrol (MG)	Model: human colonic epithelial cells NCM460	Mogrol alleviated ulcerative colitis by promoting AMPK activation	[148]
Fisetin	Model: male C57BL/6J mice and RAW264.7 macrophages	Fisetin mitigated hepatic ischaemia–reperfusion injury by regulating the GSK3β/AMPK/NLRP3 inflammasome pathway	[149]
Nitazoxanide and tizoxanide	Model: wild-type C57BL/6J mice, ApoE−/− mice and RAW264.7 macrophages	Nitazoxanide and tizoxanide inhibited the activation of the NLRP3 inflammasome in macrophages through the AMPK/IκBα/NF-κB pathway. Nitazoxanide inhibited the formation of atherosclerotic plaques in ApoE−/− mice fed a Western diet	[150]
Galanin	Model: male C57BL/6 mice and the murine macrophage cell lines J774A.1 (ATCC TIB67) and RAW264.7	Galanin ameliorated liver inflammation and fibrosis in mice by activating AMPK/ACC signalling and modifying the inflammatory phenotype of macrophages	[151]
Metformin	Model: male C57BL/6J ApcMin/+ mice and the human monocytic myeloid cell line THP-1	Metformin-induced activation of AMPK decreased the abundance of MDSCs and M2 macrophages by downregulating the mevalonate pathway	[135]
Calycosin-7-glucoside (CG)	Model: male db/db mice (age, 6–8 weeks) and RAW264.7 macrophages	Calycosin-7-glucoside promoted the polarisation of M2 macrophages and accelerated the healing of diabetic wounds in db/db mice through the ROS/AMPK/STAT6 pathway	[152]
Oleoylethanolamide	Model:mmacrophages derived from THP-1 cells	Oleoylethanolamide stabilised atherosclerotic plaques by regulating macrophage polarisation via the AMPK–PPARα pathway	[153]
Bushen Huoxue decoction	Model: RAW264.7 cells (FH0328) and THP-1 cells (FH0112)	Bushen Huoxue decoction suppressed the M1 polarisation of macrophages and prevented LPS-induced inflammatory bone loss by activating the AMPK pathway	[154]
Saxagliptin	Model: diabetic rats (STZ) and human THP-1 monocytes	Saxagliptin regulated M1/M2 macrophage polarisation via the CaMKKβ/AMPK pathway to alleviate non-alcoholic fatty liver disease (NAFLD)	[155]
Polo-like kinase 1 (PLK1)	Model: ApoE−/− mice and THP-1 macrophages	PLK1 inhibited lipid accumulation in macrophages and mitigated atherosclerosis by promoting ABCA1- and ABCG1-dependent cholesterol efflux via the AMPK/PPARγ/LXRα pathway	[156]
Empagliflozin	Model: male C57BL/6J mice (weight, 20–25 g)	Empagliflozin significantly ameliorated NAFLD-related liver injury by enhancing hepatic macrophage autophagy via the AMPK/mTOR signalling pathway and inhibiting IL-17/IL-23 axis-mediated inflammatory responses	[157]
Impressic acid (IPA)	Model: murine RAW264.7 macrophages	IPA attenuated LPS-induced inflammatory responses by activating the AMPK/GSK3β/Nrf2 axis in macrophages	[158]
Salicylates	Model: murine RAW264.7 macrophages	Salicylates ameliorated dextran sodium sulphate-induced colitis by activating AMPK targeting the β1 subunit in macrophages	[159]
Gallic acid (GA)	Model: the human hepatoma cell line HepG2, murine hepatoma cell line Hepa1-6 and murine RAW264.7 macrophages	GA inhibited lipid accumulation via the AMPK pathway and suppressed apoptosis and macrophage-mediated inflammation in hepatocytes	[160]
IL-37	Model: six-week-old female C57BL/6 mice (infected with *S. japonicum*)	IL-37 alleviated hepatic granuloma caused by *Schistosoma japonicum* infection by promoting the expression of phosphorylated AMPK in macrophages and inducing M2 polarisation	[161]
5-Amino-1-β-d-ribofuranosyl-imidazole-4-carboxamide (AICAR)	Model: male C57BL/6 mice and bone marrow-derived macrophages (BMMs)	AMPKα2 subunit directly contributed to AICAR-induced macrophage polarisation at the inflamed site of the paw. AICAR reduced peripheral inflammation by inducing macrophage polarisation	[162]
Irisin	Model: murine RAW264.7 macrophages	Irisin-induced M2 polarisation enhanced osteogenesis in osteoblasts; this effect might be associated with AMPK activation	[163]
Geniposide combined with notoginsenoside R1 (GN combination)	Model: eight-week-old male ApoE−/− mice (n = 10)	GN combination attenuated inflammation and apoptosis in atherosclerosis via the AMPK/mTOR/Nrf2 signalling pathway	[164]
Adiponectin (APN)	Model: Sprague-Dawley pups	APN ameliorated GMH-induced brain injury by regulating M1/M2 microglia polarisation via the adipoR1/APPL1/AMPK/PPARγ signalling pathway in neonatal rats	[165]

## Summary and outlook

Macrophages are innate immune cells with a dynamic range of reversible activation states, including the classical pro-inflammatory (M1) and alternative anti-inflammatory (M2) phenotypes. Metabolism in macrophages is closely related to immune-related diseases, infectious diseases, cancer progression and immunotherapy. Owing to the functional plasticity of macrophages, manipulating their phenotype is considered a potential therapeutic strategy. AMPK not only acts as an energy sensor but also regulates multiple aspects of cell metabolism. In particular, activation of the AMPK signalling pathway is necessary for the effective transformation of proinflammatory (M1) macrophages to anti-inflammatory (M2) macrophages. AMPK promotes the anti-inflammatory function of macrophages and is involved in regulating their phagocytic function. Therefore, it plays an essential role in macrophage-related diseases. Owing to its significant role in metabolism, AMPK has been widely investigated as a therapeutic target in various metabolic diseases. In-depth research on AMPK activators has provided novel insights into the treatment of metabolism-related diseases and the development of novel drugs [19, 166]. In this review, we summarised the role of AMPK in macrophage polarisation in inflammation, atherosclerosis, insulin resistance and cancer. We discussed the mechanisms through which macrophages regulate their phenotypic transformation via AMPK or other pathways, which are key to understanding inflammation and developing treatment strategies for related diseases. Several studies have demonstrated that small-molecule AMPK modulators can be used in the treatment of some macrophage-related diseases. For instance, metformin can control diabetes by upregulating AMPK to reduce blood glucose levels [101]. PXL70 is a direct activator of AMPK, which can be directly activated in vitro through a conformational mechanism mediated by binding to the AdaM site. In a phase 1b study, PXL70 inhibited liver adipogenesis and improved glucose metabolism and insulin sensitivity in patients with non-alcoholic fatty liver disease. This is also the first direct AMPK activator reported to have an impact on humans in patients with metabolic diseases [41]. Activation of AMPK signalling has been shown to be beneficial in many diseases; however, contradictory evidence has been reported in animal models of inflammatory bowel disease (IBD) [74]. Due to the pro-cancer or anti-tumor effects of AMPK responding to changes in tumor microenvironment components, the regulatory role of AMPK in cancer remains controversial [128]. And the subtypes of AMPK are expressed differently in various cancers. Depending on the tumor microenvironment, these subtypes can manifest as tumor suppressors or promoters. In addition, AMPK is a regulator of autophagy, and excessive activation of AMPK signalling may induce autophagic cell death instead of survival-promoting autophagy [101]. So we need to consider the subtypes of AMPK activation, targeting specific tissues, and moderate activation, especially in the development of AMPK modulators for the treatment of macrophage related diseases. As mentioned earlier, different subunits have different subcellular localization in cells. To avoid potential adverse factors, utilizing the specificity of AMPK isomers and the differences in distribution of different subtypes to selectively develop AMPK activators that can target specific tissues may be an effective strategy for treating macrophage related diseases. For example, when treating muscle related diseases, AMPKγ3 selective activators can target and limit the activation of AMPK on skeletal muscle, thereby reducing the risk of adverse effects of AMPK activation on other tissues and cell types [19]. Future studies should focus on investigating the actual role of AMPK in the pathophysiological processes of macrophage-associated diseases while minimising the risk of potential adverse effects. We hope to develop novel superior AMPK modulators for treating macrophage-related diseases.

## Data Availability

Not applicable.
